# Historic First Global Status Report on Drowning Prevention Highlights Challenges and Opportunities for Preventing Drowning Among Children and Adolescents

**DOI:** 10.1111/jpc.70057

**Published:** 2025-04-11

**Authors:** Sanghamitra Pati, Arohi Chauhan, Puspa Raj Pant, Bhagabati Sedain, Amy E. Peden

**Affiliations:** ^1^ Indian Council of Medical Research New Delhi India; ^2^ South Asian Institute of Health Promotion Bhubaneswar India; ^3^ Nexus Institute of Research and Innovation, Unit for Prevention and Life Saving Activities (NPLSA) Lalitpur Nepal; ^4^ Tribhuvan University, Padmakanya Multiple Campus Kathmandu Nepal; ^5^ School of Population Health UNSW Sydney Sydney New South Wales Australia

**Keywords:** adolescence, child health, education, injury, policy

**Abbreviations**
GSRDP: Global Status Report on Drowning Prevention
WHO: World Health Organisation

1

Drowning disproportionately impacts children and young people around the world. The historic inaugural Global Status Report on Drowning Prevention (GSRDP) sheds further light on this preventable cause of injury‐related mortality and morbidity [1].

Children under 15 years of age account for 43% of the global drowning burden, with 130 000 deaths in 2021. Globally, drowning is the fourth leading killer of 1–4 year‐olds and the third leading killer of 5–14 year‐olds (Table 1) [1]. In the World Health Organisation (WHO) South‐East Asian region, drowning is the third leading cause of death of children aged 5–14 years, and the 0–4 year age group records the third highest drowning rate in the world) [1]. Many countries, including India and Nepal, are facing growing drowning risks due to changing interaction with water and the increasing impacts of disasters. Even in high‐income countries, such as Australia and the United States, drowning fatalities are on the rise in recent years [2, 3].

**TABLE 1:** Number, rate and rank of drowning deaths among children 0–4 years and 5–14 years globally.

	Global	
	0–4 years	5–14 years
Number of deaths	73 000	57 000
Rate/100 000 population	10.9	4.2
Rank in leading causes of death	4	3

While effective drowning prevention interventions exist for all ages, there are several specific to children [4]. These include awareness raising campaigns and community‐level interventions such as daycare services for pre‐school children, swimming and water safety training for school aged children and the installation of barriers near water.

However, global implementation remains limited. The Status Report indicates only 28% of the 139 member states who participated reported having day‐care services for pre‐school children [1]. This is despite the well‐documented dual benefits of day‐care‐reducing drowning risk through active supervision, while simultaneously supporting early childhood development [5]. For some countries, there remain challenges to implementation due to resource constraints, administrative hurdles, and cultural factors hindering implementation, in particular in rural areas where drowning risk is higher, yet community‐based efforts show promise [6].

The GSRDP indicates implementation is slightly higher for swimming and water safety training for school‐aged children, at 28% of member states [1]. Despite the provision of technical guidance from the WHO, the unique challenges to implementation in low‐resource settings, where the drowning burden is greatest, remain [4, 7]. India reports limited subnational coverage of swimming and water safety education in the school curriculum, with a lack of awareness, standardised protocols, and policy prioritisation acting as key barriers to wider implementation, as well as a need for swimming skills training and safe places within which to conduct lessons [8]. In Nepal, efforts are underway to address the near total absence of water safety education in schools at the municipal level through sub‐national and localised drowning prevention strategies, supported by the community, yet resourcing remains a persistent challenge [9]. Despite the presence of a National Swimming and Water Safety Framework [10], recent research has highlighted significant concerns about the swimming and water safety skills of Australian children and adolescents [11].

Further, despite the high drowning burden among adolescents globally [1], there remains no high‐quality evidence on effective interventions to prevent drowning among this age group with which to guide donors and policy makers [12]. The absence of water safety education, limited awareness, and inadequate access to survival training leave adolescents particularly vulnerable [11], highlighting the need for targeted prevention measures within health and education frameworks.

Although data on child and adolescent drowning are particularly compelling to governments and donors, accurate and timely data on drowning remain a challenge in many countries. Reported drowning fatalities in India in 2021 as per data from the National Crime Bureau are 34% lower than the WHO's Global Health Estimates [1]. Similarly, Nepal's reported fatalities as sourced from the Nepal Police were 46% lower. Further investment is required to strengthen national civil registration and vital statistics systems before developing cohesive drowning surveillance systems to determine the true burden. A long‐term commitment to high quality drowning data in Australia has supported the implementation and evaluation of a range of drowning prevention interventions, as well as public communication, advocacy and policy change [13].

Underpinning these challenges is the difficulty in securing political leadership on the issue. Despite drowning being widely acknowledged as a multi‐sectoral issue [1] with relevance to many political agendas including education, gender, and climate, finding a lead ministry remains a stumbling block. India and Nepal struggle for political commitment and investment for this leading killer of children and young people, with neither country identifying a National Focal Point for Drowning Prevention in the GSRDP [1]. Although both countries include drowning prevention in strategic documents, India in its National Strategy for the Prevention of Unintentional Injuries [14] and Nepal in its Act Relating to Children [15], implementation would benefit from supporting policy instruments and frameworks, as well as dedicated resourcing.

Alongside the many challenges, there remains optimism and incredible opportunity to save hundreds of thousands of young lives across the next decade. In the South‐East Asian region, there has been a 48% reduction in all‐age drowning rates since 2000 [1], while initiatives such as pool fencing legislation have resulted in a 63% reduction in drowning deaths among children 0–4 years in Australia [16]. It is hoped the Global Status Report on Drowning Prevention will act as a catalyst for further action on this preventable killer and serve as a benchmark for which we can assess our efforts. We cannot let another report pass without meaningful action, particularly in countries with the greatest burden.

## 2 Conflicts of Interest

The authors declare no conflicts of interest.
